# Diabetes, Antidiabetic Medications and Cancer Risk in Type 2 Diabetes: Focus on SGLT-2 Inhibitors

**DOI:** 10.3390/ijms22041680

**Published:** 2021-02-07

**Authors:** Mariusz Dąbrowski

**Affiliations:** College of Medical Sciences, University of Rzeszów, Al. Rejtana 16C, 35-959 Rzeszów, Poland; mdabrowski@ur.edu.pl

**Keywords:** metformin, insulin, GLP-1 receptor agonists, DPP-4 inhibitors, thiazolidinedions, sulfonylureas

## Abstract

In the last decade, cancer became the leading cause of death in the population under 65 in the European Union. Diabetes is also considered as a factor increasing risk of cancer incidence and mortality. Type 2 diabetes is frequently associated with being overweight and obese, which also plays a role in malignancy. Among biological mechanisms linking diabetes and obesity with cancer hyperglycemia, hyperinsulinemia, insulin resistance, increased levels of growth factors, steroid and peptide hormones, oxidative stress and increased activity of pro-inflammatory cytokines are listed. Antidiabetic medications can modulate cancer risk through directly impacting metabolism of cancer cells as well as indirectly through impact on risk factors of malignancy. Some of them are considered beneficial (metformin and thiazolidinedions—with the exception of bladder cancer); on the other hand, excess of exogenous insulin may be potentially harmful, while other medications seem to have neutral impact on cancer risk. Inhibitors of the sodium-glucose cotransporter-2 (SGLT-2) are increasingly used in the treatment of type 2 diabetes. However, their association with cancer risk is unclear. The aim of this review was to analyze the anticancer potential of this class of drugs, as well as risks of site-specific malignancies associated with their use.

## 1. Introduction

In the last decade, cancer became the leading cause of death in the population under 65 in the European Union, accounting for 33% of deaths among men and 49% of deaths among women in this age group (cardiovascular diseases accounted for 24% of and 16% of deaths respectively) [1]. Diabetes in the 21st century reached epidemic level, and according to International Diabetes Federation (IDF) data, as of 2019, diabetes affects 463 million people worldwide, and its prevalence in developed countries ranges from 4% to >12% [2].

The beginnings of the scientific interest in the interrelationships between neoplasms and diabetes date back to the turn of the 19th and 20th centuries [3,4,5,6]. Tuffier was first, who described in 1888 more frequent than casual co-existence of diabetes and cancer and suggested that cancer preceded diabetes [3]. In the first period, the main attention was drawn to the relationship between diabetes and pancreatic cancer [7,8,9]. The following years of the last century brought contradictory observations concerning the higher incidence of cancer in people with diabetes, either confirming the existence of such a relationship [10,11], or not finding it [12,13]. The authors of reviews summarizing the state of knowledge at that time did not draw clear conclusions regarding the relationship between diabetes and cancer risk; however, they put forward some important suggestions for the directions of further epidemiological research, raising the role of hyperglycemia and insulin in the development of cancer [14,15]. It is worth noting here that the definition of diabetes and its diagnostic criteria have changed over the years and the current criteria were adopted by WHO (World Health Organization) in 1999 [16]. The methods and effectiveness of diabetes therapy and monitoring have also changed over the years, which has extended the life span of patients and also extended the time window for the development of malignant cancers in this population. Therefore, only the recent epidemiological data, published in this century, has given a more complete picture and documented a significantly higher incidence of certain cancers in people with diabetes, as well as with obesity [17,18,19,20]. In the last years, also the problem of oncological safety as well as oncoprotective properties of antidiabetic medications has become the subject of extensive discussion.

The aim of this review, representing mainly a clinician’s point of view on this problem, is to analyze the complex relationship between diabetes, antidiabetic medications use and cancer development, both at the epidemiological and the molecular level, with a special focus on sodium-glucose cotransporter-2 (SGLT-2) inhibitors, which becoming more widely used in the recent years.

## 2. Causes of Increased Cancer Incidence and Mortality in People with Diabetes

There are at least several hypothetical mechanisms linking diabetes with an increased risk of cancer. Both of these diseases share common risk factors, such as poor diet, low physical activity, and obesity, frequently associated with excess of abdominal fat. They can lead to the development of metabolic abnormalities, such as insulin resistance, hyperinsulinemia, increased levels of Insulin-like Growth Factor-1 (IGF-1), an increase of the concentration of steroid and peptide hormones and an increase of the activity of pro-inflammatory cytokines. All these factors, together with hyperglycemia, play a key role in the carcinogenesis in people with diabetes mellitus. In a recently published review, Vincent & Yaghootkar concluded, on the basis of numerous genetic and epidemicological studies, that increased cancer risk in type 2 diabetes is not driven by shared genetic etiology but by impaired metabolic traits associated with type 2 diabetes. Contribution of these traits is not equal, with the highest role of adiposity and hyperinsulinemia and the lowest contribution of hyperglycemia [21]. Surely, each site-specific cancer has its own, distinct pathophysiological pathways leading to its initiation, development, and progression; however, many parts of this process are common for all malignant cancers.

### 2.1. Growth Factors

The role of hyperinsulinemia in the cancer progression has been documented both in experimental studies as well as in epidemiological observations. As early as 1970, Takizawa et al. demonstrated that insulin causes an increase in DNA synthesis in the culture of breast cancer cells, both hormone-dependent (enhancing the effects of prolactin and diethylstilbestrol) and autonomous [22]. Heuson and Legros, while administering glucose and insulin to rats with a breast tumor, observed a greater increase in tumor mass compared to the control group, especially under the influence of insulin. The greatest acceleration was observed with the combined administration of glucose and insulin [23].

Epidemiological evidence for the role of hyperinsulinemia in neoplastic diseases was provided by Kaaks et al., who proved that elevated C-peptide levels were associated with a significant increase in the risk of colorectal cancer in women [24]. A similar relationship for this neoplasm in men was demonstrated by Ma et al. [25]. Pisani in her meta-analysis showed that increased levels of C-peptide or endogenous insulin are associated with an increased risk of pancreatic, colorectal, and breast cancer, but not endometrial cancer. The author also showed a relationship between hyperglycemia and pancreatic and colorectal cancer [26].

**Figure 1:**
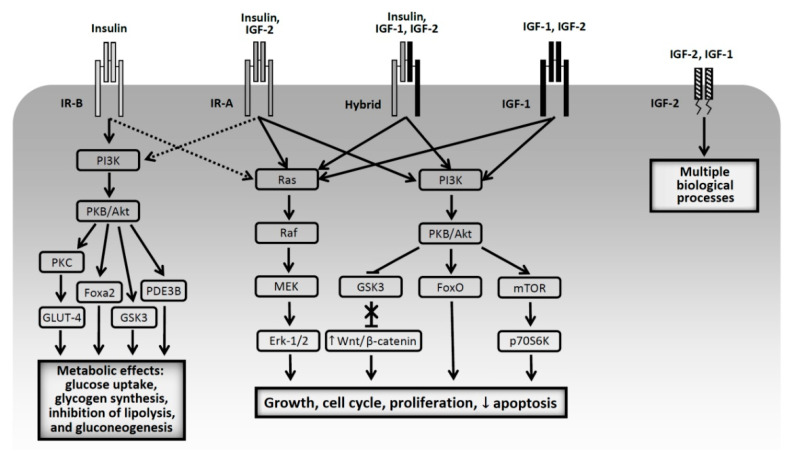
Schematic presentation of types of insulin and IGF receptors and post-receptor effects after binding to their ligands (based on [34,35,36,37,38,40]). PI3K—phosphatidylinositol 3-kinase; PKB—protein kinase B; PKC—protein kinase C; Fox—forkhead box protein; PDE3B—phosphodiesterase 3B; GLUT-4—glucose transporter-4; GSK3—glycogen synthase kinase 3; ERK—extracellular signal-regulated kinases; mTOR—mammalian target of rapamycin, p70S6—p70S6kinase. Solid lines: main pathways activated by ligand binding, dotted lines: additional pathways.

Last decades have brought a lot of data documenting the relationship between activation of insulin receptors (IR), IGF receptors and carcinogenesis. Frasca et al. demonstrated increased expression of the insulin receptor isoform A (IR-A) in breast, colorectal and lung cancer cells. IR-A, compared to IR-B, has a higher affinity for IGF-2 [27]. A similar pattern was found by Vella et al. in the case of thyroid cancer [28]. While the role of IGF-1 in carcinogenesis is established [29,30,31,32], the role of the insulin receptor is still not fully recognized. IR-A plays a special role, stimulation of which preferentially activates pathways stimulating cell growth and proliferation and inhibiting their apoptosis, while stimulation of IR-B activates mainly metabolic pathways [33,34,35,36]. Regulation of the insulin receptor isoforms expression is complex and still not fully elucidated. In this process transcription factors, splicing factors and several miRNAs may play an important role. Moreover, binding of different ligands to IR-A isoform (apart of insulin and IGF-2, also proinsulin and IGF-1—in high concentration—have an affinity to IR-A), may be associated with differences in intracellular signaling pathways and with different biological effects, which is discussed in more details in a review by Belfiore et al. [36]. IR-A is overexpressed not only in solid tumors (liver, breast, colon, endometrial, ovarian, prostate, bladder, thyroid, clear cell renal cell carcinoma, but also in multiple myeloma. However, also IR-B and IGF-1 receptors were overexpressed on the surface of cancer cells, especially in clear cell renal cell and hepatocellular carcinoma (IR-B) and in breast, ovarian, prostate, head and neck, squamous lung cancer and melanoma (IGF-1 receptor) [36,37,38]. A more recent research showed that in Drosophila melanogaster the insulin receptor substrate chico regulates insulin production and plays a role in tumor-suppressive cell competition. Its downregulation leads to hyperinsulinemia and, through drosophila insulin-like protein 2 (Dilp2), activates mTOR and induces tumorigenesis of oncogenic scribble (scrib) cells by allowing them to avoid epithelial cell competition. Metformin, by inhibiting mTOR, suppresses induced by hyperinsulinemia scrib cells proliferation. As the authors concluded, this in vivo observation provides mechanistic link between metabolic disease (e.g., diabetes) and cancer risk via systemic regulation of cell competition [39]. The pathophysiological pathways linking endogenous hyperinsulinemia with cancer are discussed in details in a recently published review paper by Gallagher and LeRoith [40]. The individual types of receptors, the post-receptor pathways they activate and the ligands that bind to them are schematically illustrated in Figure 1.

The association of IR with oncogenesis is supported by the fact that cancer cells with a smaller number of insulin receptors proliferated more slowly in culture, and implanted to mice were characterized by slower growth, lower angiogenesis and lymphogenesis, as well as created fewer metastases in the lungs and liver compared to cells with the correct expression of the receptor [41]. In addition, a diet with calorie restriction, leading to a decrease in endogenous insulin and IGF-1 levels, reduces the stimulation of mitogenic pathways, reducing the risk of cancer [42]. Ketogenic diets, through decreasing glucose and insulin levels, also exerted anti-tumor effect which was documented by Klement in his review [43]. Thus, insulin resistance, with the accompanying hyperinsulinemia, hyperglycemia, and other features of metabolic syndrome, acts as a mitogen by binding IRs on cancer cells, moreover, it also leads to increased IGF-1 synthesis in the liver and suppresses IGF binding proteins, which increases the bioavailability of this growth factor [40,44,45].

### 2.2. Adiposity and Inflammation

Epidemiological evidence is linking obesity with at least 13 cancer sites, which was documented in two large meta-analyses [20,46]. The pathomechanisms linking obesity and diabetes with cancer risk include obesity-associated dysfunction of adipose tissue, which play an important endocrine and metabolic role [46]. Its excess, due to its hyperplasia and hypertrophy, leads to impaired secretion and action of hormones and cytokines produced by adipose tissue. The production of resistin, leptin, PAI-1 (plasminogen activator inhibitor-1) and VEGF (vascular endothelial growth factor) as well as inflammatory cytokines: TNF-α (tumor necrosis factor-α), IL-6 (interleukin-6), IL-1β (interleukin 1β), MCP-1 (monocyte chemoattractive protein-1) and CRP (C-reactive protein) increases, while production of adiponectin, which improves insulin sensitivity, inhibits angiogenesis, protects against inflammation and which is also (in vitro) an inhibitor of cell growth and proliferation of certain cancers, decreases. Inflammatory cytokines also activate the NF-κB (nuclear factor kappa B) transcription factor that plays an important pro-proliferative role [45]. In recent years, a number of newer adipokines involved in inflammatory process in adipose tissue were also identified: calprotectin, CHI3L1/YKL40 (chitinase-3-like protein 1), IL-32 (interleukin-32), osteopontin, chemerin, tenascin C, visfatin and WNT-5A (wingless-type MMTV integration site family 5A). All these adipokines play an important role in insulin-resistance, type 2 diabetes and also cancer development [46]. Moreover, a part of adiponectin, also a downregulation of other anti-inflammatory adipokines: fibroblast growth factor 21 (FGF21), secreted frizzled-related protein 5 (SFRP5) and lipocalin 2 (LCN-2) is observed [47].

The excess of adipose tissue also causes increased expression of aromatase—an enzyme that converts androgens to estrogens—and insulin resistance results in a reduced production of sex hormone binding globulin (SHBG), which leads to an increase in the bioavailability of estrogens. The estrogen receptor interacts with IGF-1 and insulin receptors in activating the MAPK (mitogen-activated protein kinase) pathway, thereby stimulating the proliferation of neoplastic cells [48]. This association is reflected by a significant reduction of the risk of cancer following bariatric surgery, which was driven mainly by the reduction of endometrial and breast cancer, and it was observed predominantly in women [49,50,51].

### 2.3. Hyperglycemia

Hyperglycemia in an experimental study by Heison and Legros accelerated the development of breast cancer in rats [23]. Epidemiological studies published in the first decade of 21st century, involving different populations, also showed an association between glycaemia and cancer risk. Such a relationship has been demonstrated for fasting blood glucose [52,53,54,55,56] as well as for glucose load [53]. HbA_1c_ levels also correlate with cancer risk in most studies [57,58,59,60,61]. This relationship was particularly evident in studies conducted solely in the population of diabetic patients [58,59,60], where it was linear from HbA_1c_ > 7.0% (53 mmol/mol). In a systematic review of 19 studies published in 2016, increasing level of HbA_1c_ was robustly associated with increasing risk of colorectal, pancreatic, respiratory and uterine cancers, while for other site-specific cancer such associations were inconsistent [62]. However, some studies did not find such an association between long-term glycemic control and cancer risk [63].

The pathomechanisms linking hyperglycemia with increased cancer risk are complex. The direct effects of high glucose concentration include induction of mutations, effects on proliferation, and activation of invasiveness and migration of tumor cells. Hyperglycemia causes an overproduction of reactive oxygen species (ROS) in the mitochondrial electron transport chain. This leads to the activation of, among others, advanced glycation end-products (AGE) pathway [64]. By binding to a specific RAGE receptor (receptor for AGE), AGE activates the NF-κB transcription factor, which results in the formation of free oxygen radicals inside cell nuclei. This leads to DNA damage, acting mutagenically [50,65]. High glucose concentrations may alter the expression of certain genes (including cyclin A and E), thus affecting the cell life cycle, and activate the Wnt/β-catenin signaling pathway, resulting in increased proliferation of tumor cells. Finally, hyperglycemia increases the mobility of tumor cells by increasing its invasiveness through E-cadherin inhibition and activation of PKCα [66]. Cancer cells, unlike nonproliferating cells, switch their metabolism from oxidative phosphorylation to oxygen glycolysis, known as the Warburg effect. They consume the vast majority of glucose to produce lactate, which allows the synthesis of sufficient amounts of amino acids, nucleotides and lipids for cell replication at the cost of ATP production (this process produces only four moles of ATP from one mole of glucose). Neoplastic cells tolerate an acidic environment, so the presence of lactic acid may promote tumor growth. As it results, with the availability of the energy substrate, which is glucose, almost all activity of neoplastic cells is directed towards proliferation and increasing biomass [66,67]. This may explain the accelerated proliferation of these cells under hyperglycemia [23], but also the inhibited tumor growth under fasting conditions [42,68].

**Figure 2:**
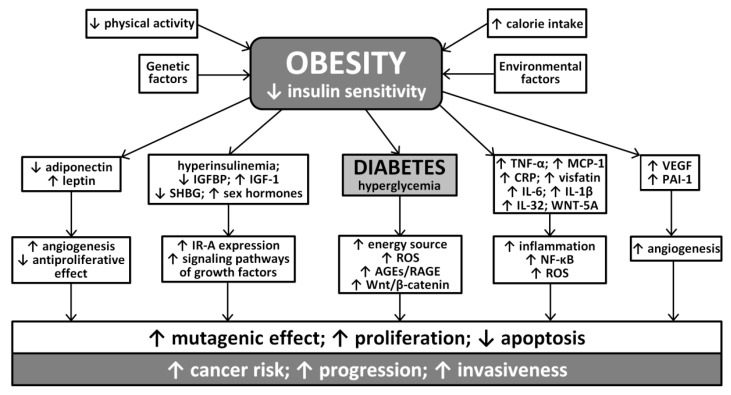
A simplified view of the relationship between obesity, diabetes and related metabolic disorders and cancer risk (based on [29,30,31,32,33,34,35,36,37,43,44,45,46,47,48,63,64,65,66]). IGFBP—insulin-like growth factor binding protein; SHBG—sex hormone binding globulin; VEGF—vascular endothelial growth factor; PAI-1—plasminogen activator inhibitor-1; TNF-α—tumor necrosis factor-α; MCP-1—Monocyte Chemoattractive Protein-1, CRP—C-reactive protein; IL-6—interleukin-6; IL-1β—interleukin 1β; IL-32—interleukin-32; IR-A—insulin receptor A; ROS—reactive oxygen species; AGE—advanced glycation end-products; RAGE—receptor for AGE; NF-κB—nuclear factor kappa B.

Schematic, simplified relationship between hyperglycemia, obesity, insulin resistance, hyperinsulinemia and inflammation, and the increased incidence of cancer in the diabetic population is schematically presented in Figure 2.

## 3. Antidiabetic Medications and Cancer

Due to its progressive nature, type 2 diabetes requires a gradual intensification of treatment, starting usually with monotherapy, through the use of 2–3 oral medications, up to injectable therapies with the use of GLP-1 (Glucagon-like peptide-1) receptor agonists and/or various insulin therapy regimens, with or without oral medications [69,70]. Therefore, it is not easy to determine the individual effect of particular antidiabetic drugs on the risk of cancer, due to the long time of cancer development and the changing therapeutic models over time [71]. Moreover, an interplay between antidiabetic medications, can also have an influence on cancer risk. Nevertheless, many data, mainly from observational studies (randomized clinical trials are usually too short to draw firm conclusions on the risk of cancer), indicate the protective effect of metformin and the pro-oncogenic effect of exogenous insulin. According to current data, other classes of drugs may carry a risk of site-specific cancers, but longer follow-up is necessary to confirm or rule out such relationships.

### 3.1. Metformin

The first clinical reports indicating the protective effect of metformin in terms of cancer incidence and cancer death risk appeared in the middle of the first decade of this century [72,73]. Own studies also confirmed protective effect of metformin in the Polish population, irrespective of gender [74,75]. In numerous meta-analyses of case-control and cohort studies, metformin use was associated with 10–40% reduction of cancer incidence and mortality, which was summarized by Heckman-Stoddard et al. in their paper [76]. Schulten in his review documented beneficial effect of metformin in many site-specific cancers. Metformin was associated with lower incidence of gastric, colorectal, liver, breast and endometrial cancers, and with longer survival/lower mortality from colorectal, lung, breast, endometrial, prostate and pancreatic cancers [77].

One of the mechanisms of the antihyperglycemic action of metformin is inhibition of the activity of complex I of the mitochondrial respiratory chain, which reduces the availability of ATP and inhibits gluconeogenesis. This process requires the activation of AMP-activated protein kinase (AMPK). Phosphorylation of AMPK occurs in the presence of LKB1 (Liver kinase B1), a tumor suppressor gene product, and is facilitated by AMP. Activation of AMPK leads to the inhibition of the mTOR (mechanistic target of rapamycin) signaling protein. The second site of antihyperglycemic action of metformin is gut, where metformin increases both glucose utilization as well as lactate production by enterocytes, which contributes considerably in maintaining glucose homeostasis [78].

However, inhibition of mTOR leads not only to inhibition of gluconeogenesis in hepatocytes, but also reduces protein synthesis and inhibits the proliferation of neoplastic cells, inducing their apoptosis and cycle-cell arrest [79,80]. Anti-tumor effect of metformin was also documented by Sanaki et al. [39]. AMPK-dependent pathway is considered to play important role in anticancer effect of metformin in several site-specific cancers, e.g., breast [81], endometrial [82] or colorectal cancers [83]. Moreover, this mechanism also plays a role in mitigating DPP-4 inhibitor-induced breast cancer metastasis [84].

Metformin can also inhibit cancer growth indirectly. Decreased hepatic glucose production and increased peripheral glucose uptake, mainly by muscle cells, lead to reduced insulin release from pancreatic β-cells and decreased plasma insulin level, thus reducing risk of proliferation of neoplastic and pre-neoplastic cells [79]. Schulten in his review also discusses several other pathways leading to decreased cancer risk associated with metformin. Metformin inhibits transforming growth factor beta 1 (TGF-β1) thus leading to inhibition of epithelial-to-mesenchymal transition (EMT); it also inhibits cyclin D1 leading to cell cycle arrest; it inhibits AKT serine/threonine kinase 1 leading to growth inhibition; it promotes immune response through protection of CD8+ tumor infiltrating lymphocytes and accumulation of M1-like macrophages which reduces cancer growth and angiogenesis; it inhibits signal transducer and activator of transcription 3 (p-STAT 3) thus activating apoptotic and autophagous pathway through downregulating BLC 2, apoptosis regulator; it also upregulates some micro RNAs which inhibits cancer cells growth and impairs cancer cells viability; it inhibits vascular endothelial growth factor (VEGF) thus inhibiting angiogenesis; it also leads to cancer stem cells depletion through different signaling pathways. Metformin can also inhibit mTOR pathway through DNA damage inducible transcript 4 (DDIT 4) thus inhibiting protein synthesis in cancer cell. Moreover, metformin combined with other chemotherapeutic drugs demonstrates additive or synergistic effect to enhance its anticancer action [77].

### 3.2. Sulfonylurea Derivatives

Drugs of this class, by binding to a specific sulfonylurea receptor 1 (SUR1) on the β-cell surface, cause exocytosis of insulin accumulated in secretory granules [85]. This leads to an increase in endogenous insulin levels and may theoretically be associated with an increased risk of cancer. The first observational studies seemed to confirm this relationship [72,73,86], although there were differences between individual formulations [87]. Own observation, as well as the largest systematic review, did not show an increased risk of cancer associated with the use vs. non-use of sulfonylureas [74,75,88].

### 3.3. Insulin

The first observation of an association between the use of exogenous insulin and the risk of colorectal cancer dates back to 2004 [89]. Subsequent studies have confirmed a higher incidence of cancer and a higher risk of death from cancer in people using insulin [72,73,86]. The controversial (due to the methodology used) work by Hemkens et al. showed a higher risk of cancer in patients using long-acting insulin analog glargine compared to human insulin. However, it also indicated a much more significant dose-dependent increase in the risk of cancer regardless of the type of insulin used [90]. In the following years, many studies analyzing the relationship between exogenous insulin and cancer were published. A meta-analysis performed by Karlstad et al. revealed a significant 52% increase in cancer risk in people treated with insulin [91]. Insulin monotherapy was associated with elevated risk of cancer incidence in a dose-dependent manner in a study by Holden et al. [92]. Own study also showed elevated risk of cancer associated with insulin monotherapy, but it was attenuated to insignificant level when insulin was combined with metformin [93]. On the other hand, in prospective, randomized clinical trial (RCT), Outcome Reduction with an Initial Glargine Intervention (ORIGIN) trial, elevated cancer risk among insulin users has not been found [94]. However, in this study relatively low doses of insulin were used, while in a real-life setting, insulin therapy is usually initiated in patients with longer-lasting diabetes, worse metabolic control and in older age compared to patients treated with other therapeutic regimens. Frequently, large doses of insulin are used to attain and maintain glycemic control. Such a cluster of risk factors of malignancy can significantly influence cancer risk. Thus, due to a mitogenic effect of exogenous insulin in a mechanism similar to endogenous insulin, its overdosing, generating iatrogenic hyperinsulinemia, should be avoided.

### 3.4. Thiazolidinedions

Agonists of the γ isoform of the peroxisome proliferator-activated receptor gamma (PPAR-γ), a nuclear hormone receptor, are a group of antidiabetic drugs that have been present on the market for a several years. Their main site of action is adipose tissue, where they improve insulin sensitivity and inhibit the production of pro-inflammatory cytokines [95]. Their multiple side effects (weight gain, increased risk of heart failure, edema and bone fractures) limit their use in therapy. In the meta-analysis of 119 studies use of glitazones was associated with significant reduction of overall cancer risk [96]. Glitazones exerts its anticancer effect in several mechanisms: cell growth arrest through decreasing activity of S-phase kinase-associated protein 2 (Skp2), a ubiquitin ligase, which leads to upregulation of p27^kip1^, a cyclin-dependent kinase inhibitor; induction of apoptosis through upregulation of genes p53, PTEN and BAX (BCL2 Associated X), apoptosis regulator, and downregulation of bcl-2/bcl-xL and survivin, antiapoptotic molecules; and inhibition of invasion by inhibition of MEK/ERK signaling pathway which leads to increased expression of E-cadherin and claudin-4 [97]. However, in people using pioglitazone, a higher risk of bladder cancer was found in meta-analysis of RCTs and observational studies. In case of observational studies this association was time- and dose-dependent. It is hypothesized by the authors, that this phenomenon is associated with high expression of PPAR-γ in bladder cells in which their activation is associated with tumor growth and progression [98]. A lower dose of pioglitazone seems to have less adverse effects, while not compromising its beneficial effects on blood glucose, insulin sensitivity and cancer [97].

### 3.5. Dipeptidil-Peptidase-4 Inhibitors

Dipeptidil-peptidase-4 (DPP-4) is an enzyme involved in degradation of many substrates including growth factors, chemokines, neuropeptides, vasoactive peptides and incretin hormones-glucagon-like peptide-1 (GLP-1) and glucose-dependent insulinotropic polypeptide (GIP). GLP-1 and GIP by binding to specific receptors on the surface of pancreatic β-cells, stimulate insulin release in response to meal, mainly reach in carbohydrate content. Thus, inhibition of their degradation by DPP-4 inhibitors increases incretin hormones activity and has an impact on glucose metabolism which is used in type 2 diabetes treatment [99]. The animal studies suggested that inhibition of DPP-4 may be associated with elevated risk of pancreatic, ovarian, prostate, skin, and lung cancers [100]. Moreover, it was also postulated that DPP-4 inhibition can increase the risk of metastasis through induction of CXCL12/CXCR4, which activates mTOR to promote epithelial–mesenchymal transition (EMT) [101]. On the basis of the US Food and Drug Administration’s database of adverse event reporting system (AERS), based on voluntary reports sent by physicians, Elashoff M et al. in 2011, revealed increased risk of pancreatitis and pancreatic cancer associated with the use of sitagliptin, a DPP-4 inhibitor [102]. These data raised concerns about DPP-4 inhibitors long-term safety. However, later years did not confirm these findings. Meta-analysis of site-specific cancers associated with DPP-4 inhibitors use in 12 RCTs and 13 observational studies did not reveal elevated cancer risk in DPP-4 inhibitors’ users [103]. The most recent meta-analysis of 157 RCTs, performed by Dicembrini and colleagues, revealed neutral effect of DPP-4 inhibitors on overall cancer risk, irrespective of molecule studied and cancer site, with the exception of colorectal cancer, in which DPP-4 inhibitors use was associated with its significantly reduced risk [104].

### 3.6. Glucagon-Like Peptide-1 Receptor Agonists

Similar to sitagliptin, Elashoff M et al. revealed elevated risk of pancreatitis, as well as pancreatic and thyroid cancers in patients treated with exenatide, a GLP-1 receptor agonist (GLP-1 RA) [102]. These findings also raised concerns regarding safety of this class of drugs. In fact, stimulation of the GIP and GLP-1 receptor, apart from insulinotropic effects, also activates anti-apoptotic and pro-proliferative processes. The final effect of their stimulation is the activation of the pathway leading to the activation of Mek 1/2 (MAPK/ERK kinase 1/2) and ERK 1/2 (extracellular signal-regulated kinase), which, after entering to the nucleus, catalyzes the phosphorylation of transcription factors leading to cell proliferation and differentiation. It also inhibits the activity of caspase-3, an enzyme involved in the apoptotic process. Activation of GIP and GLP-1 receptors stimulates the proliferation of not only β-cells but also their progenitor cells. In islet cell culture, both GIP and GLP-1 induced the transcription of the cyclin D1 gene, an enzyme that is crucial for the initiation of mitosis in most cell types. In addition, activation of the canonical β-catenin/Wnt signaling pathway was observed [105,106,107]. Moreover, GLP-1 receptors are also present in many other tissues, including thyroid, exocrine pancreas, liver, hypothalamus, renal tubules, and bone, and their activation produces effects completely unrelated to glucose homeostasis [102].

Further observations did not confirm elevated risk of cancer in GLP-1 RA users. Cao C et al. in the most recent meta-analysis of the 37 RCTs did not reveal elevated risk of pancreatic, thyroid and overall cancer in GLP-1 RA users compared to controls [108].

### 3.7. Sodium-Glucose Cotransporter-2 Inhibitors

Different types of sodium-glucose cotransporters are present in the entire body. Sodium-glucose cotransporter-2 (SGLT-2) is mainly present in the S1 segment of the proximal convoluted tubule in the kidney (but it was also found in the pancreas, brain, liver, thyroid, muscle and heart). Its main role is reabsorption of glucose from glomerular filtrate [109]. SGLT-1 is also present in the kidney (in the S3 segment of the proximal convoluted tubule), but the main location of its action is the small intestine [109,110]. SGLT-2 and SGLT-1 play important role in maintaining glucose homeostasis. Inhibition of SGLT-2 leads to increased glucose excretion with urine and offers glucose lowering effect independent of insulin [110]. SGLT-2 inhibitors in the CardioVascular Outcome Trials (CVOTs) and renal outcome trials showed, in addition to glucose-lowering effect, body weight and blood pressure reduction, a wide pleiotropic effect, resulting in a reduction of the risk of cardiovascular events, heart failure and kidney disease progression [111,112,113,114,115,116,117,118,119,120]. This resulted in far-reaching changes in scientific associations’ clinical practice recommendations and enabled a wide entrance of these compounds to the market of antidiabetic medications [69]. However, at the beginning of SGLT-2 inhibitors use, concerns were raised about an increased risk of bladder and breast cancers, which led to the rejection of an application for approval of dapagliflozin by Food and Drug Administration in 2011 [121]. In addition, early meta-analysis performed by Vasilakou et al. revealed imbalanced incidence of bladder and breast cancer in dapagliflozin users compared to controls [122]. More recent meta-analysis of 46 RCTs performed by Tang et al. did not reveal overall increased cancer risk. However, it maintained concerns about an increased risk of bladder cancer. On the other hand, the total number of cases (18/22,359 vs. 1/12,228) was too low to draw far-reaching conclusions. Interestingly, canagliflozin use was associated with lower risk of gastrointestinal cancers, which was not observed for dapagliflozin and empagliflozin [123]. The most recent meta-analysis of 27 RCTs performed by Dicembrini et al. did not reveal any difference in cancer incidence between SGLT-2 inhibitors and comparators, including placebo [124].

**Figure 3:**
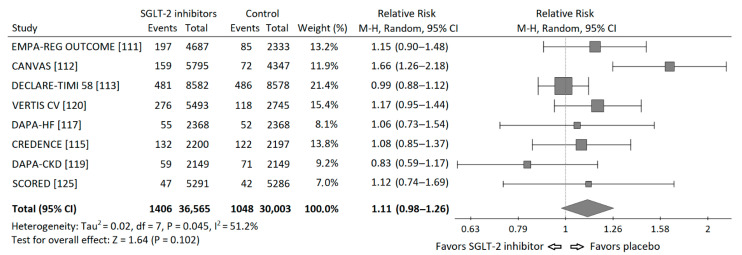
Forest plot of meta-analysis of the risk of neoplasms incidence for SGLT-2 inhibitors vs. placebo in cardiovascular and renal outcome trials. The size of the square box is proportional to the weight that each study contributes in the meta-analysis. The overall estimate and CI are marked by a diamond.

Based on the data from published cardiovascular and renal outcome trials [111,112,113,114,115,116,117,119,120,125], I performed a meta-analysis of the risk of neoplasms incidence in those trials. I requested the data regarding incidence of malignancies in the Empagliflozin Outcome Trial in Patients with Chronic Heart Failure and a Reduced Ejection Fraction (EMPEROR Reduced), but I did not receive these data and it was not included into meta-analysis. Statistical analysis was performed using PQStat v.1.8.0 software (PQStat Software, Poznań, Poland). A random effects model (Mantel-Haenszel method) was used to calculate pooled Risk Ratios (RR). Overall risk of neoplasm incidence appeared to be insignificant, HM-RR 1.11 (95% confidence interval 0.98–1.26), *p* = 0.102. The RRs for each study and their summary are presented as the forest plot (Figure 3).

A moderate but significant heterogeneity between the studies was found, I^2^ = 51.2%, Q-statistics 14.35, *p* value 0.045. It is worth to note, that there were important differences between populations included in particular studies. In the Dapagliflozin in Patients With Heart Failure and Reduced Ejection Fraction (DAPA-HF) trial exclusively patients with heart failure and reduced ejection fraction (EF) below 40% were included [116], while in the renal outcome trials: Canagliflozin and Renal Events in Diabetes With Established Nephropathy Clinical Evaluation (CREDENCE) and Dapagliflozin and Prevention of Adverse-outcomes in CKD (DAPA-CKD), patients had chronic kidney disease (CKD) mainly at stages 3 and 4 of (⅔ and 90% respectively) [114,118]. Moreover, the duration of each study was different (from 1.2 to 4.2 years). In addition, in DAPA-HF and DAPA-CKD trials also patients without diabetes were included. Thus, the risk of neoplasm incidence was different in particular studies, which can, at least partly, explain the significant heterogeneity found in the analysis.

There are several mechanisms linking SGLT-2 inhibitors with cancer risk. Glucose is the main source of energy for cancer cells. It enters into the cell by using glucose transporters, mainly GLUT 1, which are frequently overexpressed in cancer cells. However, also presence or overexpression of sodium-glucose cotransporters have been identified on the surface of several site-specific cancer cells. Both SGLT-1 and SGLT-2 are present in pancreatic, brain and prostate cancers, in addition SGLT-1 is present in ovary, head and neck cancers, while SGLT-2 was found in the lung, breast and liver cancers [126,127]. Thus, inhibition of SGLT-2 may have a direct impact on cancer cells growth. Studies in vitro and in animal models documented such effect of dapagliflozin in case of CaKi-1 renal cell cancer line with high expression of SGLT-2 [128], canagliflozin in two cell lines of hepatocellular cancer [129], ipragliflozin, canagliflozin and dapagliflozin in breast cancer [130,131].

The metabolic effect associated with SGLT-2 inhibitor use include lowering of blood glucose level, weight reduction, mainly fat mass, and decreased endogenous insulin secretion or decreased exogenous insulin demand and improved peripheral insulin sensitivity, i.e., they have a positive impact on factors associated with cancer risk. In addition, positive effect associated with SGLT-2 inhibitors use is observed in nonalcoholic fatty liver disease (NAFLD), which is considered as a risk factor of cirrhosis and hepatocellular carcinoma [110]. Thus, SGLT-2 inhibitors can also act on cancer cells indirectly. Jojima et al. demonstrated significantly lower steatosis score in mice treated with canagliflozin compared to vehicle and, in long-term observation, significantly fewer hepatic tumors in the group using continuously canagliflozin compared to the vehicle group. Moreover, canagliflozin in vitro attenuated HepG2 cells proliferation via activation of caspase-3 [132]. Canagliflozin also appeared to be effective in inhibiting lung and prostate cancer cells growth in vitro via inhibiting mitochondrial complex-I supported respiration [133]. In the mouse models of obesity-related cancers: breast and colon cancer, canagliflozin slowed tumor growth. This effect was abrogated by insulin infusion. Thus, in case of obesity-related cancers canagliflosin exerts its oncoprotective effect through insulin- and glucose-lowering action [134]. In a study by Shiba et al. canagliflozin attenuated development of HCC in a mouse model of human NASH. Administration of canagliflozin was associated with improvement in hyperglycemia, hyperinsulinemia, inflammation in liver and adipose tissue, liver steatosis and reactive oxygen species generation, which led to decreased risk of HCC development [135]. Saito et al. demonstrated significant reduction in a number of HCT116 colon cancer cells associated with dapagliflozin treatment, and this effect was independent of SGLT-2 inhibition [136]. Kato et al. revealed in diabetic, obese mice significant suppression of the development of colorectal neoplastic lesions after administration of tofogliflozin. This effect was not associated with direct inhibition of SGLT-2, but with glucose-lowering effect and amelioration of chronic inflammation [137].

On the other hand, also increased risk of certain cancers during the use of SGLT-2 inhibitor was described. Rats treated for two years with canagliflozin at dose 100 mg/kg. showed increased incidence of pheochromocytomas and renal tubular tumors, while testicular Leydig cell tumors were observed also with doses 10 and 30 mg/kg [138]. However, further study revealed that these tumors were secondary to malabsorption of glucose, and were not due to a direct effect of canagliflozin [139]. Moreover, the highest dose of canagliflozin was over 20-fold higher than it is used in clinical practice.

Long-term data from human studies assessing impact of SGLT-2 inhibitors on cancer are scarce. In a population-based cohort study conducted by Suissa et al. with the use of the U.K. Clinical Practice Research Datalink (CPRD), no difference in breast cancer incidence between new SGLT-2 and DPP-4 inhibitors users was found. However, median follow-up was only 2.6 years, thus, SGLT-2 inhibitors might not exert beneficial effects associated with metabolic changes associated with their use [140]. García et al. analyzed association of SGLT-2 inhibitors use and bladder cancer reported in the European Pharmacovigilance Database. They found significantly higher number of cases associated with SGLT-2 inhibitors use compared to other antidiabetic medications, after exclusion of pioglitazone [141]. However, this can be the case similar to association of sitagliptin and exenatide with pancreatic cancer reported by Elashoff M et al. in 2011 [102], which was not confirmed in further observations.

## 4. Discussion

In the recent years, both obesity and diabetes became highly prevalent worldwide [2,142]. Both of them are associated with elevated risk of cancer incidence and mortality [17,18,20]. Antidiabetic medications can modulate this risk via different mechanisms. The aim of this review was to analyze how different classes of antidiabetic drugs influence cancer risk. For me, as a clinician, it was important how the data from randomized trials and observational studies on the relationship between diabetes treatment and cancer risk could be explained by basic science at the molecular level, and how it can be translated back to the clinical practice. Obviously, clinical observations are not free from different biases. Due to the fact that diabetes treatment regimen changes over time with diabetes duration, several time-related biases—immortal time bias, time window bias and time lag bias can occur, which was described by Suissa in 2012 on the example of observational studies with metformin [143]. These biases can lead to overestimation of the impact of antidiabetic medications on cancer risk. However, in the case of metformin, clinical observations of its beneficial effect on cancer were supported by basic science. Molecular studies revealed several mechanisms, a part of antihyperglycemic effect of metformin, in which this drug exerts its anticancer effect. This, in turn, reassured clinicians to use this drug in their everyday clinical practice and the position of metformin in clinical practice recommendations of medical societies is not endangered [69,70]. This case underlines the important role of bilateral communication and interaction between clinical practice and basic science. In addition, in case of other antidiabetic medications, clinical observations can be explained at the molecular level, and then it can be translated back to real-life practice. For example, based on clinical observations and the known molecular mechanisms of insulin action, the benefits and risks of initiating and continuing insulin therapy in terms of cancer risk should always be considered. In addition, surely, heroic doses of insulin should be avoided [89,90,91,92,93,94]. The same applies to glitazones (in the Polish market only pioglitazone remained in use). The lower doses are associated with lower risk of adverse effects with almost unaffected benefits in terms of improving hyperglycemia and insulin sensitivity, and lowering cancer risk in both direct and indirect mechanisms [95,96,97]. However, in case of thiazolidinediones, it is important to be aware of the increasing risk of bladder cancer with the increasing duration of exposure to pioglitazone [98]. The associations between incretin-based therapies and cancer are still not fully elucidated. Although there are some concerns regarding DPP-4 inhibitors and breast cancer and its metastasis risk, it seems that both DPP-4 inhibitors and GLP-1 receptor agonists can be considered neutral, or even beneficial (decreased risk of colorectal cancer in case of DPP-4 inhibitors) [99,100,101,102,103,104,105,106,107,108]. Hopes associated with GLP-1 receptors agonists use in terms of cancer risk were not confirmed, despite their beneficial effect on glycemia, body weight, blood pressure, liver steatosis and insulin sensitivity. Moreover, some uncertainties with regards to pancreatic cancer still remain [102,108].

The main focus in my review was on SGLT-2 inhibitors, a new class of antidiabetic medications with a proved, impressive cardiovascular and renal benefits regardless of their impact on glycemia [111,112,113,114,115,116,117,118,119,120]. Due to their mechanism of action—improvement in hyperglycemia, insulin sensitivity, decreased body weight—there were also expectations for their beneficial effect on cancer risk. In vitro and animal studies show their significant potential for protection against cancer formation and progression [126,127,128,129,130,131,132,133,134,135,136,137]. However, randomized and observational studies, and their meta-analyzes, did not confirm these hopes [111,112,113,114,115,116,117,118,119,120,121,122,123,124,125]. Moreover, data from the European Pharmacovigilance Database again raised concerns regarding elevated bladder cancer risk in SGLT-2 inhibitors users in real-life setting [141].

## 5. Materials and Methods

The number of literatures regarding associations between diabetes and cancer can be considered almost unlimited. The phrase “diabetes and cancer” typed in the search box in Pub-Med gives over 67,000 results. In my review, I tried to find the most recent, the most relevant and the most important papers in the field (e.g., highly cited). In the case of medications other than SGLT-2 inhibitors, I relied mainly on data from meta-analyzes of randomized trials and observational studies, supported by the molecular studies of their mechanisms of action. In the case of SGLT-2 inhibitors, the data were taken from CVOTs and renal outcome trials, and they were the source of meta-analysis which I performed. I took also into account meta-analyzes of randomized trials and observational studies on the relationship between SGLT-2 inhibitors use and risk of malignancy. Finally, I analyzed in more details data from in vitro and animal studies supporting conception of protective effect of SGLT-2 inhibitors on cancer risk.

## 6. Conclusions

In summary, antidiabetic medications apparently differ in their impact on the risk of cancer. Metformin and thazolidinediones can be considered beneficial (with the exception of bladder cancer in the latter case); incretin drugs seem to be neutral, although they have potential for benefits, especially GLP-1 receptor agonists; while insulin, due to its mitogenic effect, should be used with caution. The question regarding if SGLT-2 inhibitors have anticancer potential or if they are potentially harmful is still unanswered. Obviously, RCTs last too short to exert all beneficial metabolic (decreased body weight, improved insulin sensitivity) and anti-inflammatory effects of SGLT-2 inhibitors in terms of cancer risk. Thus, long-term observations are required to demonstrate how SGLT-2 inhibitors use influence the risk of malignancy. Hopefully, future well-designed prospective studies and analyses based on large databases will give, at least partly, an answer regarding lowered or increased risk of different site-specific cancers associated with SGLT-2 inhibitors use.

## Data Availability

Not applicable.
